# Tailoring femtosecond 1.5-μm Bessel beams for manufacturing high-aspect-ratio through-silicon vias

**DOI:** 10.1038/srep40785

**Published:** 2017-01-18

**Authors:** Fei He, Junjie Yu, Yuanxin Tan, Wei Chu, Changhe Zhou, Ya Cheng, Koji Sugioka

**Affiliations:** 1 RIKEN Center for Advanced Photonics, Hirosawa 2-1, Wako, Saitama 351-0198, Japan; 2 State Key Laboratory of High Field Laser Physics, Shanghai Institute of Optics and Fine Mechanics, Chinese Academy of Sciences, P.O. Box 800-211, Shanghai 201800, China; 3 Laboratory of Information Optics and Optoelectronic Technology, Shanghai Institute of Optics and Fine Mechanics, Chinese Academy of Sciences, P.O. Box 800-211, Shanghai 201800, China; 4 School of Physical Science and Technology, Shanghai Tech University, Shanghai 200031, China

## Abstract

Three-dimensional integrated circuits (3D ICs) are an attractive replacement for conventional 2D ICs as high-performance, low-power-consumption, and small-footprint microelectronic devices. However, one of the major remaining challenges is the manufacture of high-aspect-ratio through-silicon vias (TSVs), which is a crucial technology for the assembly of 3D Si ICs. Here, we present the fabrication of high-quality TSVs using a femtosecond (fs) 1.5-μm Bessel beam. To eliminate the severe ablation caused by the sidelobes of a conventional Bessel beam, a fs Bessel beam is tailored using a specially designed binary phase plate. We demonstrate that the tailored fs Bessel beam can be used to fabricate a 2D array of approximately ∅10-μm TSVs on a 100-μm-thick Si substrate without any sidelobe damage, suggesting potential application in the 3D assembly of 3D Si ICs.

Since the invention of the integrated circuit (IC) in 1958, silicon etching has been a crucial process in the semiconductor industry, and substantial effort has been directed toward increasing the degree of integration of ICs. Recently, to achieve higher integration and operating speed, three-dimensional (3D) Si ICs based on 3D assembly have received much attention. At present, one of the key technologies in the 3D assembly of Si ICs is the manufacturing of through-silicon vias (TSVs), which are electrical connections that run vertically through Si substrates to interconnect Si IC chips. Compared with the package-on-package technology, TSVs allow 3D ICs to be assembled with higher density and shorter connection length, resulting in a smaller footprint as well as higher performance^1^,^2^,^3^.

The present requirements for TSVs include a diameter of ~50 μm, a depth of ~500 μm, and an aspect ratio of ~10. It remains a significant challenge to develop high-throughput and cost-effective TSV fabrication techniques for mass production that allow for high-aspect-ratio etching, controllable morphology, and low loading effects. Additionally, downsizing of the TSV diameter while maintaining the aspect ratio is a requirement for future 3D ICs.

The typical TSV fabrication techniques currently in use include wet chemical etching and plasma dry etching^4^,^5^,^6^. However, the application of these techniques is limited due to the anisotropic etching rates in the monocrystalline Si substrates commonly employed in TSV fabrication, which make it intrinsically very difficult to create high-aspect-ratio structures. On the other hand, the reactive ion etching (RIE) method (most often the Bosch process) can be used to fabricate a wide variety of deeply etched Si structures with small feature sizes; however, RIE involves several steps, including photolithography, and suffers from a very low etching rate.

TSV fabrication via direct laser drilling has been proposed as a fast and environmentally friendly (free of toxic chemicals and gases) alternative, with fewer processing steps due to the elimination of photolithography. The fabrication of 20-μm-diameter holes in a 250-μm-thick Si substrate using nanosecond UV laser percussion has been reported^7^, but deep drilling of holes smaller than 10 μm in diameter remains a significant future challenge for the assembly of 3D Si ICs. Recently, ultrafast laser processing has been proven to be an attractive tool for material processing, as it allows for sub-diffraction-limit processing with suppression of the heat-affected zone^8^,^9^,^10^,^11^,^12^,^13^,^14^,^15^,^16^,^17^,^18^,^19^. For example, the high-quality cutting of planar silicon devices using 780-nm femtosecond (fs) lasers has been successfully demonstrated^19^. In addition, high-speed and high-aspect-ratio drilling of through-holes in various materials using ultrafast laser processing has been a hot topic in many important scientific and industrial applications. Femtosecond Bessel beams have been used to fabricate microstructures with aspect ratios of up to 10^2^–10^3^ in various silica glasses, without the need for sample translation due to the long depth of field^20^,^21^,^22^,^23^,^24^,^25^. We have demonstrated TSV fabrication by using fs Bessel beams with wavelengths tuning from 400 nm to 2.4 μm^26^. In Bessel beam processing, the material should be transparent to the laser wavelength so that the sidelobes of Bessel beam can propagate through the material to generate the central lobe in the material. Therefore, in case of Si microstructuring using Bessel beams, the wavelength of the fs laser should be longer than 1.13 μm, since the band gap of Si is 1.12 eV. However, even fs lasers operating in the transparent window of Si cannot easily perform deep etching of Si due to its narrow band gap, strong two-photon absorption, and weak free carrier absorption^27^,^28^,^29^,^30^,^31^. Moreover, it has been recently reported that there are inherent limitations for the volume and rear surface modification of Si when using 1.3-μm fs Bessel beams^30^. Therefore, deep etching of Si using transparent fs lasers is still challenging.

In this study, we propose the use of a 1.5-μm fs Bessel beam for TSV manufacturing. We first optimize the TSV fabrication conditions, including the laser energy per pulse, laser shot number, and sample position. To eliminate the ablation intrinsically induced by the sidelobes of a tightly focused fs Bessel beam and to achieve deep etching, we proposed to tailor the beam using a specially designed binary phase plate^26^. The tailored Bessel beam has a sidelobe ratio, which is defined as the ratio of the maximum peak intensity of the first-order sidelobe to that of the central lobe, of 0.6%, compared with 15.6% for a conventional fs Bessel beam generated by an axicon lens. Suppression of sidelobes was also proposed by a different concept based on interference between two Bessel beams with different wave vectors^32^. However, an annular aperture with two slits was used for this method, which significantly reduced the utilization efficiency of laser beam. Additionally, the sidelobe ratio achieved was 4.3% which is not sufficient for TSV manufacturing with no sidelobe damage as discussed later. In this paper, the specific experimental setup for TSV fabrication using the tailored Bessel beam is designed. Furthermore, details of the theoretical model and the numerical design of phase plate are discussed and presented. More importantly, fabrication of ~10-μm diameter TSV arrays with a pitch of 50 μm were, which corresponds to a TSV density of ~1 × 10^5^ cm^−2^ and an aspect ratio of ~10, is demonstrated by optimally tailoring the fs Bessel beam. Finally, we give brief discussion in terms of commercial applications of the developed technique.

## Experimental

**Figure 1:**
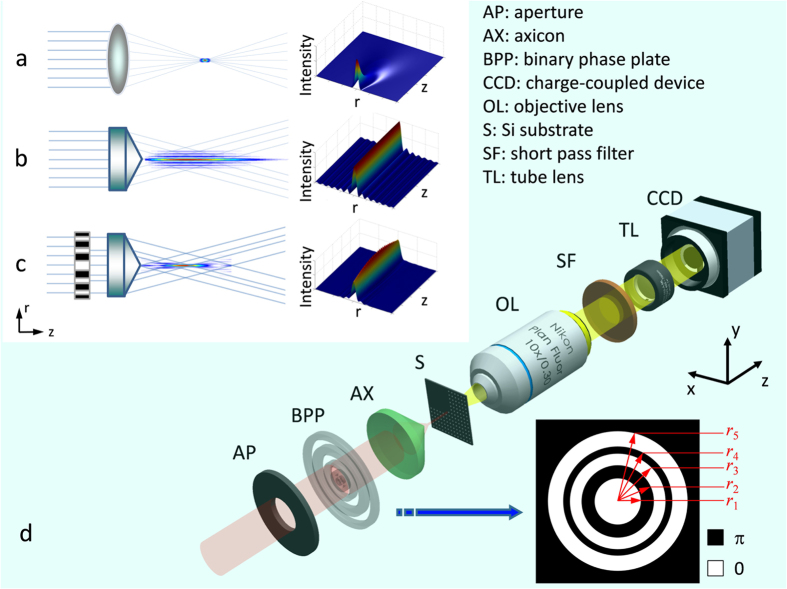
Schematic illustrations of the generation schemes and intensity profiles for a (**a**) Gaussian beam, (**b**) conventional Bessel beam, and (**c**) tailored Bessel beam, and (**d**) the experimental setup for TSV fabrication. The inset shows a sketch of the structure of a binary phase plate.

The 800-nm pulses of a 1-kHz amplified Ti:sapphire fs laser beam were converted to 1.5-μm pulses in an optical parametric amplifier (OPA). The pulse energy and duration of the 1.5-μm pulses were 550 μJ and 65 fs, respectively. In order to demonstrate the principle of the proposed technique, three different types of fs laser beams—a Gaussian beam, a conventional Bessel beam, and a tailored Bessel beam—were employed, as shown in Fig. 1(a–c), respectively. The Gaussian beam was tightly focused by an objective lens, and had a short depth of field with no sidelobes. In contrast, the conventional Bessel beam, which was produced using an axicon lens, had an extraordinarily long depth of field but a large sidelobe ratio of ~16%. In principle, there is a tradeoff between the depth of field and sidelobe ratio of laser beams due to the diffraction property of light waves. By placing an optimally designed phase plate in front of the axicon, a tailored Bessel beam with a significantly reduced sidelobe but sufficiently long depth of field can be generated for many important applications, some of which will be described below.

Figure 1(d) shows a schematic illustration of the experimental setup employed in this study. A tunable circular aperture was used to truncate the laser beam from ∅5 mm to ∅4 mm to guarantee good beam quality. In the present work, we used an axicon (AX2520-C - 20.0°, ∅25.4 mm, Thorlabs Inc.) with a 20° base angle for the generation of conventional Bessel beams. To tailor the conventional Bessel beam, the phase plate was placed 5 mm in front of the axicon. The Si sample was mounted on an XYZ-stage with a resolution of 0.3 μm. To investigate the 3D intensity distributions of each laser beam, a microscope imaging system comprising an objective lens (NA 0.3, 10x), a tube lens, and a near-infrared CCD camera was located behind the axicon and was fixed on a stage that could be translated along the propagation direction of the laser beam. To prevent laser damage to the CCD camera, a short-pass filter was inserted for attenuation of the 1.5-μm fs beam. The 3D intensity distributions of the laser beams were reconstructed by translating the microscope imaging system stepwise along the laser beam axis and recording the beam pattern at each position.

The Si samples used in this study were mirror-polished, (100)-oriented, and n-doped (resistivity ρ > 20 Ω⋅cm) substrates with thicknesses of 50 μm and 100 μm. For the TSV fabrication, the front surface of the Si samples were set at z = z_max_ (the position of the on-axis maximum intensity), the position of which was determined by examining the 3D beam profiles measured with the beam profiling system. A programmable shutter with a 1-ms time resolution was employed to vary the number of shots used in the TSV fabrication, and the laser pulse energy was adjusted with neutral density filters.

## Results

### Design, fabrication, and characterization of a binary phase plate

Currently, laser beam shaping through amplitude and/or phase modulation offers diverse features for achieving super-resolution spots, homogeneous illumination, extension of the depth of field, and multi-spot parallel processing for many important applications such as microscopy, laser micromachining, nanolithography, optical trapping, and data storage^33^,^34^,^35^,^36^. Among the various laser beam shaping optics, binary phase plates have received a great deal of interest owing to their high conversion efficiency, low cost, and ease of manufacture^37^,^38^,^39^,^40^. Using a binary phase plate, we attempt to eliminate the sidelobe ablation associated with conventional Bessel beams. The sidelobes of Bessel beam are, in principle, produced by interference of all the beams propagating along the surface of a “cone”. The effect of using phase plate is to introduce more Bessel-like beams whose sidelobes are distributed at different radii. By tuning the phase of each Bessel-like beam, one may achieve destructive interference between all the sidelobes to suppress their intensities, whereas the constructive interference of the central lobe still remain induced to achieve a high intensity of the central lobe. Such optimization is very complicated, so that self-learning with a computer simulation was employed to optimize the design of phase plate.

The structure of a typical binary phase plate is illustrated in the inset of Fig. 1(d), in which the black and white regions correspond to 0- and π-phased zones, respectively. To design a phase plate that generates the best output beam for a given set of requirements, parameters including the number of annular zones (M), the radii of each zone (r_m_, where m = 1, 2, …, M, as defined in the inset of Fig. 1(d)), and the depth of the circular grooves (Δh) should be optimized. Here, we restricted our design to a 0-π phase plate; thus, the groove depth was fixed to generate a π-phase difference, and the only parameters that required optimization were the number of annular zones and the ring radii.

An ideal Bessel beam contains infinite energy and is purely non-diffractive during propagation, but does not exist in the real world^20^,^21^. In practical applications, a Bessel-Gaussian beam can be formed as an approximation to an ideal Bessel beam by focusing a Gaussian beam with an axicon lens, as shown in Fig. 1(b). We refer to such beams as “conventional Bessel beams” in order to distinguish them from the tailored Bessel beams used in this study. For a perfect axicon, i.e., one with a perfectly sharp tip, the intensity profile of the Bessel beam can be analytically derived, as discussed elsewhere^22^.

In this work, a more practical round-tip-axicon model^22^, in which the axicon surface is considered to be a hyperboloid of revolution of two sheets, was employed to achieve an accurate phase plate design. The distance between the apex of the hyperboloid and that of an ideal axicon was measured to be τ = 21 μm (measured with an optical microscope; see Fig. S1 in the Supporting material for more detail). To design a proper phase plate for tailoring fs Bessel beams, a numerical method based on Hankel transformations was adopted to simulate the beam propagation after the axicon and the phase plate (see Methods). At each iteration of the simulation, a simulated annealing (SA) algorithm^41^ was adopted to optimize the parameters (M, {r_m_}) of the phase plate (see Fig. S2). We chose this method because it provided the best fit for the optimization of multiple variables as well as a good balance between the rate of convergence and optimization time. By setting the length of the searching window to 100 μm in the simulation, the optimized radii of each zone of the phase plate were obtained as r_m_ = {146, 185, 200, 1696, 1920} μm.

**Figure 2:**
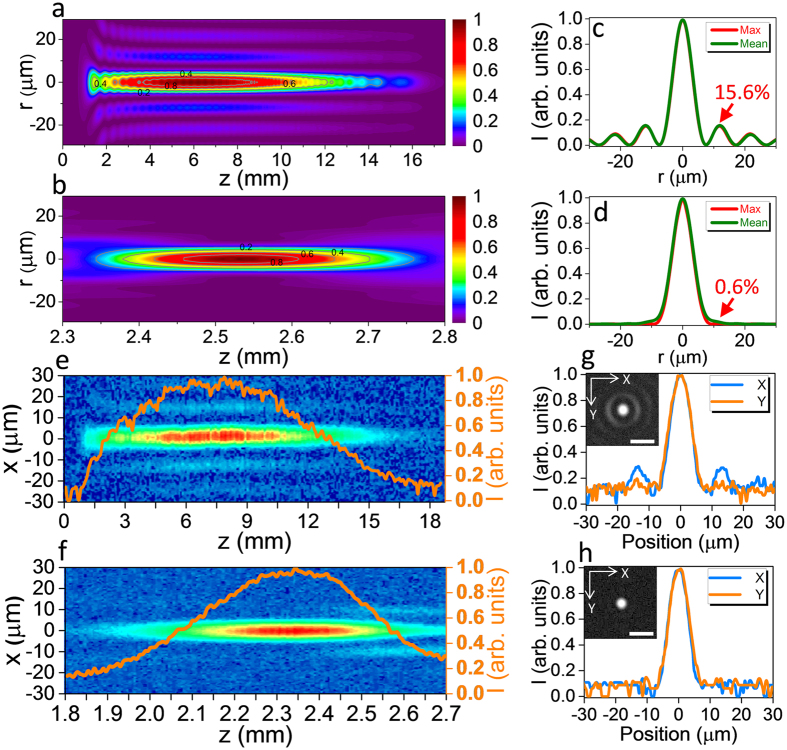
(**a**–**d**) Calculated and (**e**–**h**) experimentally measured intensity distributions of the conventional and tailored Bessel beams, respectively. (**a**) and (**b**) show the calculated beam profiles in the r-z plane of the conventional and tailored Bessel beam, respectively. Their transverse intensity profiles at z = z_max_, where the maximum intensity is obtained on the laser beam axis, and of the mean values are exhibited in (**c**) and (**d**), respectively. Experimentally measured 2D profiles of each beam in the x-y plane together with the on-axis intensity distributions (orange curve) are shown in (**e**) and (**f**), respectively. (**g**) and (**h**) show the measured transverse intensity distributions of the conventional and tailored Bessel beams at z = z_max_, respectively, along with CCD-captured images of the respective beams in the insets. The scale bars in the inset in (**g**) and (**h**) are 20 μm.

Figure 2 displays the lateral and axial profiles of the conventional and tailored Bessel beams, from which the beam waist (diameter), depth of field, and sidelobe ratio were derived, as summarized in Table 1. Based on the results, it is clear that employing an optimally designed phase plate can efficiently reduce the sidelobe ratio of a Bessel beam. Compared with the conventional Bessel beam, the depth of field of the tailored Bessel beam is shorter; however, it is still sufficiently long for TSV fabrication and other applications. It should be emphasized that in Fig. 2, in order to make an intuitive comparison of the side-lobe ratios of different beams, all the beam intensity profiles are normalized. In the practical case in our calculation, the peak intensity of the tailored Bessel beam becomes ~1.4 fold as that of the conventional Bessel beam. This is preferable in TSV fabrication since the necessary pulse energy for TSV fabrication will be reduced after using the phase plate as discussed later.

The designed phase plate was manufactured on a BK7 glass substrate using conventional photolithographic techniques detailed elsewhere (see Methods)^42^. We characterized the fabricated phase plate in terms of suppression of the sidelobes. Relatively low pulse energy was utilized to ensure linear beam propagation. The laser beam pattern was measured and analyzed using the beam profiling system depicted in Fig. 1(d). The intensity profile of the conventional Bessel beam is shown in Fig. 2(e). The depth of field was measured to be ~12 mm, and intensity modulation along the longitudinal direction was also observed. The results are in accordance with the theoretical design when considering the round-tip axicon lens, as shown in Fig. 2(a). The measured longitudinal profile of the tailored Bessel beam is shown in Fig. 2(f). For the tailored Bessel beam, z_max_ and z_DOF_ were evaluated to be 2.35 mm and 420 μm, respectively. More importantly, no sidelobe was observed within the depth of field. The total loss of laser power after passing through the phase plate should be mainly caused by the Fresnel reflection on both sides of the phase plate, which is estimated to be ~8%. This kind of loss can be easily reduced by AR-coating of the phase plate. Thus, the efficiency of this tailored technique is consierably high. It should be noted that the measured intensity profile of the tailored Bessel beam deviates slightly from the theoretical result shown in Fig. 2(b). This can be attributed to the relatively deteriorated quality of the 1.5-μm fs laser beam generated by the OPA compared with the Ti:sapphire fundamental, as well as the limited resolution in the fabrication of the phase plate (see Figs S3 and S4). The lateral beam profiles of the conventional and tailored Bessel beams at z = z_max_ are shown in Fig. 2(g) and (h), respectively. The sidelobes were found to be remarkably diminished by the phase plate, which is a promising result for the fabrication of high-quality TSVs.

### TSV fabrication by 1.5-μm femtosecond Bessel beams

**Figure 3:**
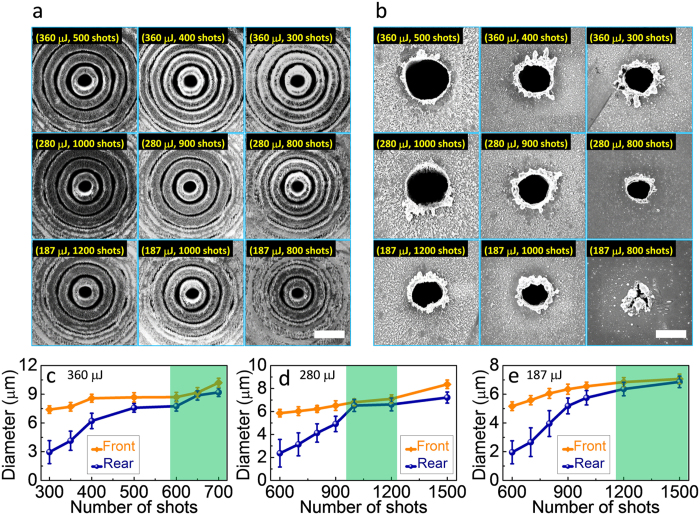
SEM images of TSVs at the (**a**) front and (**b**) rear surfaces of a 50-μm-thick Si sample fabricated using the conventional Bessel beam at different pulse energies and with different laser shot numbers. The scale bars in (**a**) and (**b**) are 20 μm and 5 μm, respectively. (**c**)–(**e**) show the variation in the diameters of the TSVs at both the front and rear surfaces with pulse energy and laser shot number.

We first performed Si drilling in air using the conventional fs Bessel beam with various pulse energies and numbers of laser shots, and with the Si samples fixed at z = z_max_. Figure 3(a) and (b) respectively show scanning electron microscopy (SEM) images of the front and rear surfaces of the TSVs fabricated in 50-μm-thick Si samples. The TSVs fabricated under these experimental conditions had diameters of 5–10 μm and aspect ratios of 5–10. The TSV diameter as a function of number of laser shots is shown in Fig. 3(c–e) for different pulse energies. Production of a single through-hole using a conventional fs Bessel beam with a pulse energy of 360 μJ required more than 300 laser shots. Interestingly, nearly taper-free TSVs could be created by increasing the number of laser shots to ~650. It was also observed that for lower pulse energies of 280 μJ and 187 μJ, the required number of laser shots to produce taper-free TSVs increased to ~1000 and ~1500, respectively. Below an energy of 187 μJ, however, through-holes could not be formed even when the laser shot number was significantly increased.

Even though taper-free TSVs could be created using the conventional Bessel beam, inherent sidelobe ablation caused by strong two-photon absorption of Si at the front surface was inevitable, as shown in Fig. 3(a). Such sidelobe ablation at the front surface of Si could not be totally suppressed even by using a moderate laser pulse energy of 187 μJ or number of laser shots, which in any case would hamper practical applications of TSVs. Further investigation revealed that through-holes could not be formed in 100-μm-thick Si substrates even when using the maximum output pulse energy (~550 μJ) of our system and increasing the number of laser shots.

TSV fabrication using a conventional Bessel beam in air suffers from two issues that must be overcome for future practical applications. One is the aforementioned sidelobe-induced damage and the other is the limited depth of the TSVs. To this end, we propose to tailor the conventional Bessel beam using an optimally designed phase plate, as discussed in Section 3. We first compared the characteristics of TSV fabrication for three different types of fs laser beams: a focused Gaussian beam, a conventional Bessel beam, and the tailored Bessel beam. For each case, we set the pulse energy to be slightly larger than the threshold of through-hole formation, and optimized the laser shot number such that the taper angle of the TSVs was minimized. During the laser processing, the Si substrates were again fixed on a stage such that the front surface corresponded with z = z_max_. In the case of the Gaussian beam, focusing with an objective lens (NA 0.3, 10x) produced an approximately ∅6 μm beam with a z_DOF_ of ~16.7 μm. The optimal pulse energy and laser shot number for the Gaussian, conventional Bessel, and tailored Bessel beams were determined to be 25 μJ and 400, 196 μJ and 500, and 47 μJ and 800, respectively. The significant reduction in pulse energy between the conventional and tailored Bessel beams, which can be attributed to the concentration of the beam into the central lobe by the phase plate, increases the process efficiency of the TSV fabrication.

**Figure 4:**
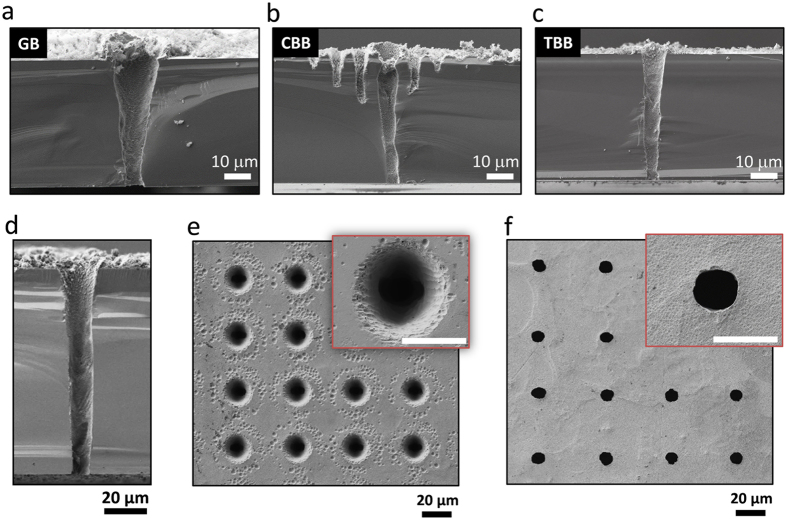
SEM images of TSVs fabricated in (**a**–**c**) 50- and (**d**–**f**) 100-μm-thick Si substrates. (**a**)–(**c**) are cross-sectional views of TSVs fabricated using a (**a**) Gaussian beam (GB), (**b**) conventional Bessel beam (CBB), and (**c**) tailored Bessel beam (TBB), respectively. (**d**)–(**f**) are cross-sectional images of the front and rear surface of TSVs produced in 100-μm-thick Si substrates by the tailored Bessel beam, respectively. The scale bars in the insets of (**e**) and (**f**) are 5 μm.

After fs laser ablation, all of the TSV samples were mechanically cut and examined. Figure 4(a–c) show SEM cross-sectional images of the TSVs created in the 50-μm-thick samples using the Gaussian, conventional, and tailored Bessel beams, respectively. The Gaussian beam clearly produced TSVs with front surface diameters of larger than 20 μm, corresponding to a low aspect ratio of less than 3, and with severely tapered sidewalls. For the conventional fs Bessel beam, high-aspect-ratio (5 μm in diameter, corresponding to an aspect ratio of ~10) TSVs with almost taper-free sidewalls were created; nevertheless, the high sidelobe ratio of the beam led to severe ablative damage surrounding the TSVs. In contrast, by employing the tailored Bessel beam, the sidelobe ablation was completely suppressed and TSVs with diameters of less than 8 μm were formed in the 50-μm-thick substrate.

We then attempted to fabricate deep TSVs in 100-μm-thick Si substrates with the same laser beams, and found that this was only possible using the tailored Bessel beam. Since the focus of the Gaussian beam was fixed at the front surface of the substrate, the laser intensity was insufficient to induce ablation at greater depths due to the defocusing of the focused laser beam. The relatively high sidelobe intensity of the conventional Bessel beam induced two-photon absorption at the Si surface, as evidenced by the sidelobe ablation that can be seen in Fig. 4(b). Therefore, the sidelobes could not penetrate deep into the Si substrate. In contrast, suppression of the sidelobes for the tailored Bessel beam prevented two-photon absorption by Si, allowing the Bessel beam to penetrate through the entire Si substrate.

In the case of TSV drilling on the 100-μm-thick Si substrate using the tailored Bessel beam, the optimal pulse energy and laser shot number were evaluated to be 104 μJ and 1200, respectively. Under these conditions, ~10-μm diameter TSV arrays with a pitch of 50 μm were formed, which corresponds to a TSV density of ~1 × 10^5^ cm^−2^ and an aspect ratio of ~10. As can be seen from the cross-sectional view shown in Fig. 4(d), no detectable sidelobe-induced damage was observed, which proves that this method is valid even for thick Si samples. In Fig. 4(d), severe deposition of debris can be seen, particularly on the front surfaces of the Si substrates. To remove this debris, the TSV samples were bathed (without ultrasonication) in an etchant mixture of 47% HF, 70% HNO_3_, and 100% H_2_O (with a volume ratio of 5:3:1) for about ~10 s, and then washed with deionized water. After this procedure, the TSV samples exhibited clean front and rear surfaces, as can be seen in Fig. 4(e) and (f).

## Discussion

We have demonstrated the first fabrication of high-aspect-ratio TSVs using a 1.5-μm fs tailored Bessel beam. The tailored Bessel beam, which was generated using a phase plate, did not produce the same sidelobe-induced damage associated with conventional fs Bessel beams, and could be used to perform deep etching. Moreover, high-density (~1 × 10^5^ cm^−2^) TSV arrays with an aspect ratio of ~10 could be produced on 100-μm-thick Si substrates using the tailored Bessel beam. Compared with other existing technologies for TSV manufacturing, our method greatly simplifies the complex procedures of TSV fabrication.

Some issues regarding the practical application of this technique to TVS fabrication remain unresolved. The biggest issue is that the throughput is still far from the industrial requirement of 1000 holes per second. Currently, several hundred to one thousand pulses are required to create a single TSV, which corresponds to 1–2 holes per second for the repetition rate of 1 kHz used in this study. Increasing the laser repetition rate to 100 kHz may increase the throughput to 100–200 holes per second without deterioration of the fabrication quality caused by heat accumulation^43^. Moreover, employing multi-beam parallel processing would further increase the throughput. To enhance the fabrication efficiency, a decrease in the pulse energy used in the fabrication is also important. The depth of field of the tailored Bessel beam employed in this study is still significantly larger than the typical thickness of Si substrates. Designing the phase plate for an optimal depth of field can reduce the pulse energy required for TSV fabrication, which is also beneficial for further reduction of the sidelobe energy. Meanwhile, although the tailored Bessel beam is uniform in the axial direction along the depth of field, in terms of the spot size, the intensity is not uniform. This is why the diameters of the TSVs are always largest at their front surfaces (the position of the front surfaces correspond to z = z_max_ during the ablation). We expect that the Bessel beam can be tailored such that the intensity in the axial direction along the depth of field is uniform by further optimizing the design of the phase plate, which would enable faster, higher resolution, and more precise TSV fabrication.

Based on the results of this study, we believe that our novel technique for TSV fabrication can be potentially applied to the industrial-scale production of 3D Si ICs. In addition, we expect such tailored Bessel beams can be implemented to improve the performance of other important applications such as bio-imaging and optical trapping^44^,^45^,^46^.

## Methods

### Characterization of the round-tip axicon

Figure S1 shows a side view of the round-tip axicon used in the experiment. The distance between the apex of the hyperboloid and that of an ideal axicon is measured to be *τ* = 21 μm. The base angle of the axicon used is *β* = 20°. The ray angle relative to the optical axis can be expressed as ref. 22


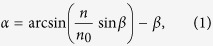


where *n* = 1.44 is the refractive index of fused silica at a wavelength of 1.5 μm and *n*_0_ = 1.

### Theory of the binary phase plate used for tailoring Bessel beams

In the simulation, the diffraction from the aperture and phase plate can be neglected, which is justified for optical elements with feature sizes >>*λ*. Moreover, a numerical method based on Hankel transformations was adopted to simulate the propagation of the beam behind the axicon and phase plate. Thus, the light field just behind a round-tip axicon (*z* = 0) is expressed as


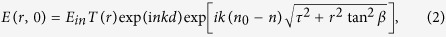


where 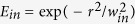 is the field of the incident Gaussian beam with a beam waist of *w*_*in*_ = 2 mm which coincides with the experimental condition, and *n* and *n*_*0*_ denote the refractive indices of the axicon and the outside medium, respectively. It should be noted that a thin axicon is assumed, with *d* the maximum (center) thickness of the axicon, and *z* = 0 defined as the apex of the corresponding ideal axicon. *T*(*r*) is the transmission function of the phase plate, and can be written as


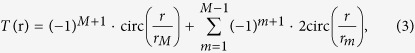


where *r*_*m*_ is the radius of the *m*th annular zone, *M* is the total number of the annular zones, and circ(·) denotes a circle function defined as


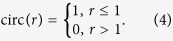


Performing a Hankel transform of *E*(*r*, 0) in a cylindrical coordinate system gives the spatial-frequency spectrum of the field at *z* = 0:


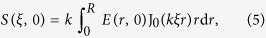


where *R* is the radius of the pupil of the axicon. We then propagate the spectrum in the spatial domain for a distance of *z* using an angular-spectrum approach^47^, and obtain


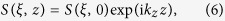


where 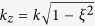 is the wave vector in the propagation direction. Thus, the field propagating a distance z after the round-tip axicon can be obtained by inverse Hankel transformation of *S*(*ξ*, *z*) to give


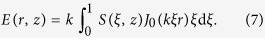


It is noteworthy that a parabolic sampling^22^ was adopted for Equations (5), (6), (7), to hasten the computing speed during the numerical simulations. To obtain the optimal structure of the phase plate, an appropriate cost function should be determined and minimized. In this work, the cost function was chosen as the average value of sidelobe ratios over a certain axial range [*z*_max_ − *l*, *z*_max_ + *l*], and is expressed as


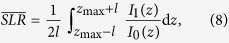


where 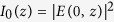 is the on-axis intensity of the beam (also the peak intensity of the central lobe), *I*_1_(*z*) is the peak intensity of the brightest sidelobe, and 2 *l* is the length of the searching window in the following optimization algorithm.

### Fabrication of the binary phase plate

For the fabrication of phase plate, BK7 glass with dimensions of 16 mm × 16 mm × 1.5 mm was chosen as the substrate. A thin layer of photoresist (Shipley S1805) was spun onto the glass substrate, and the design pattern for the phase plate was fabricated via the electron-beam lithography method. A solution of 5% HF was used to etch the substrates through the patterned photoresist for 100 s. Finally, the patterned photoresist was removed with acetone^42^.

The annular-zone structure of the phase plate was measured using a step profiler (Taylor Hobson) and a white-light interferometer (Wyko NT1000), as shown in Figures S3–S5. The average depth of the grooves was ~1.55 μm, while the theoretical groove depth to achieve a phase shift of π radians was


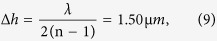


where n = 1.501 is the refractive index of BK7 at a wavelength of 1.5 μm.

## Additional Information

**How to cite this article**: He, F. *et al*. Tailoring femtosecond 1.5-µm Bessel beams for manufacturing high-aspect-ratio through-silicon vias. *Sci. Rep.* **7**, 40785; doi: 10.1038/srep40785 (2017).

**Publisher's note:** Springer Nature remains neutral with regard to jurisdictional claims in published maps and institutional affiliations.

## Supplementary Material

Supplementary Information

## Figures and Tables

**Table 1:** Comparison of beam waist of the central lobe (2w_0_, diameter measured as the FWHM), depth of field (z_DOF_, measured as the FWHM along the optical axis), sidelobe ratio at z = z_max_ (SLR_m_) and average sidelobe ratio ( 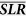 ) within the optimized axial range for the conventinal Bessel beam and tailored Bessel beam.

	2*w*_*0*_	*z*_*DOF*_	*SLR*_*m*_	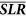
Conventional Bessel beam	6 ± 0.3 μm	12.4 ± 0.62 mm	15.6%	15.8%
Tailored Bessel beam	6 ± 0.3 μm	280 ± 14 μm	0.6%	0.7%
