# Supplementary material for: Tailoring femtosecond 1.5-μm Bessel beams for manufacturing high-aspect-ratio through-silicon vias
Source: Sci Rep. 2017 Jan 18;7:40785. doi: 10.1038/srep40785 (PMC5241677; doi:10.1038/srep40785)
Supplement: Supplementary Information [file srep40785-s1.pdf]

Supplementary Information

Tailoring femtosecond 1.5-μm Bessel beams for manufacturing high-aspect-ratio through-silicon vias

Fei He,[1,2] Junjie Yu,[3] Yuanxin Tan,[2,4] Wei Chu,[2] Changhe Zhou,[3] Ya Cheng,[2,] and Koji Sugioka[1,**]*

[*]*Corresponding author: e-mail: ya.cheng@siom.ac.cn*

[**]*Corresponding author: e-mail: ksugioka@riken.jp*

[1] RIKEN Center for Advanced Photonics, Hirosawa 2-1, Wako, Saitama 351-0198, Japan

[2] State Key Laboratory of High Field Laser Physics, Shanghai Institute of Optics and Fine Mechanics, Chinese Academy of Sciences, P.O. Box 800-211, Shanghai 201800, China

[3] Laboratory of Information Optics and Optoelectronic Technology, Shanghai Institute of Optics and Fine Mechanics, Chinese Academy of Sciences, P.O. Box 800-211, Shanghai 201800, China

[4] School of Physical Science and Technology, Shanghai Tech University, Shanghai 200031, China

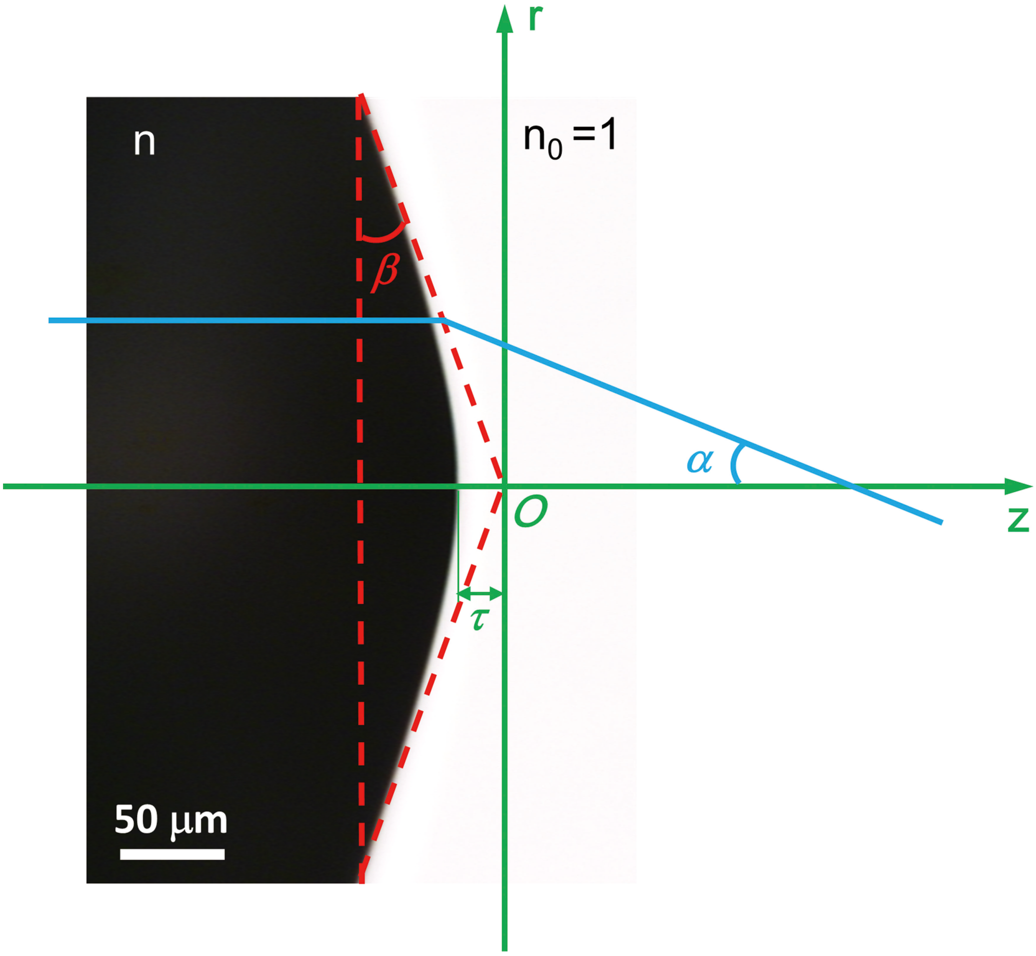

Figure S1 Optical micrograph of the round-tip axicon used for TSV fabrication.

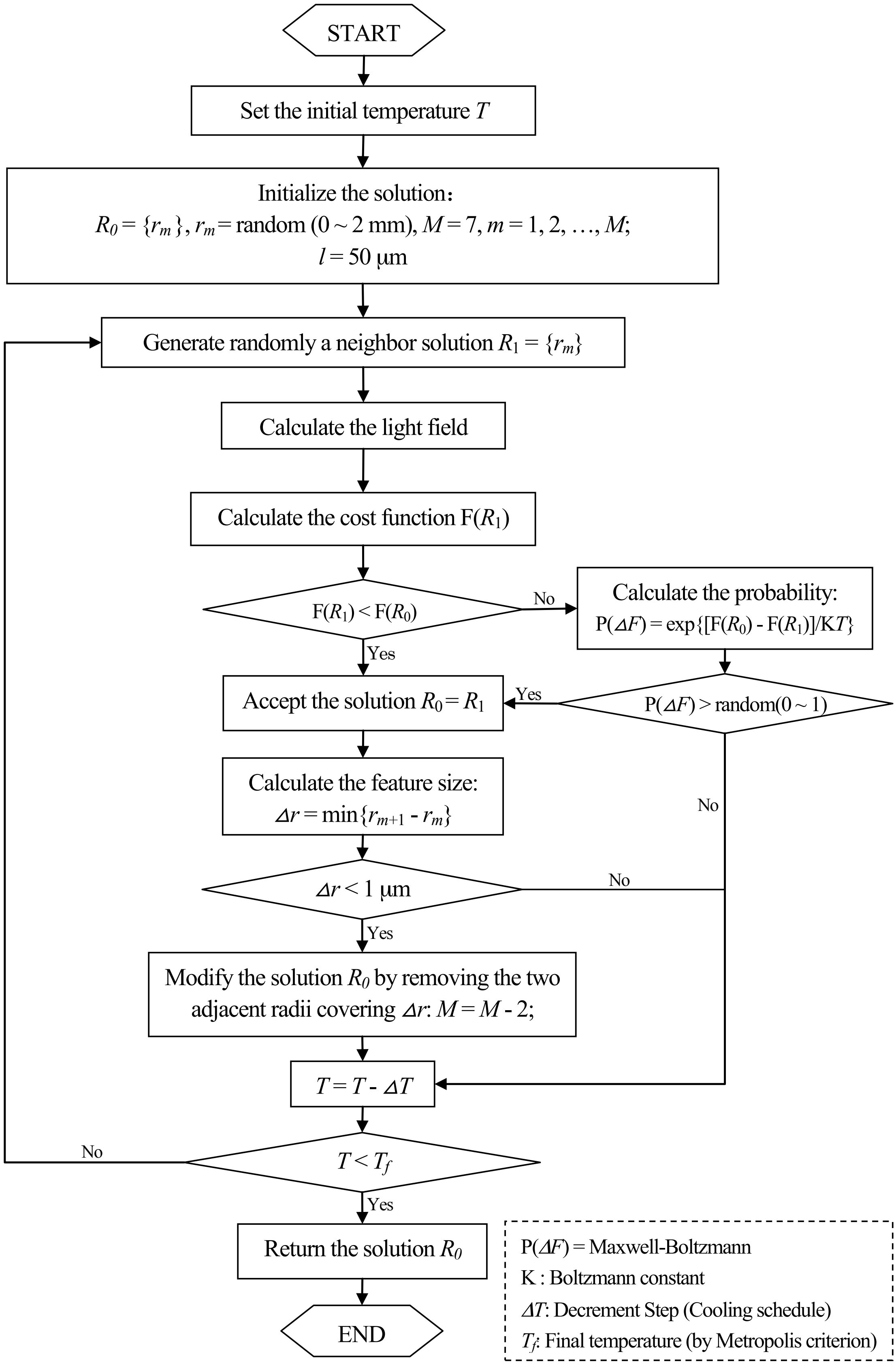

Figure S2 Flow chart of the simulated annealing (SA) algorithm.

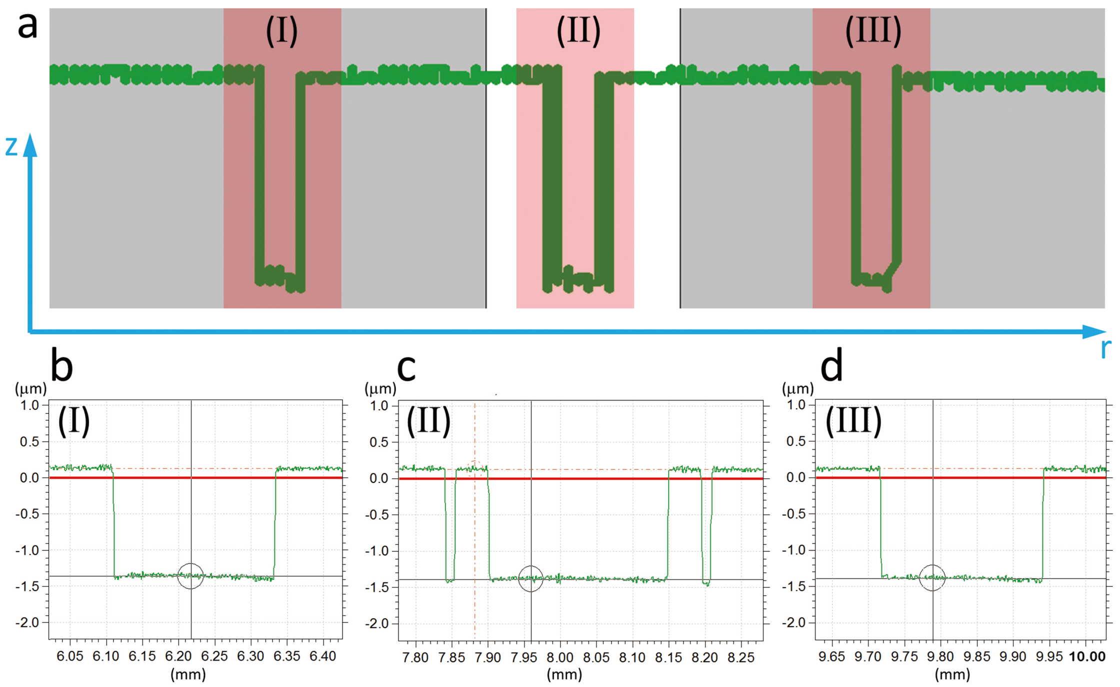

Figure S3 (a) Surface relief profile of the fabricated phase plate. Close-up views of areas I, II, and III in (a) are shown in (b), (c), and (d), respectively.

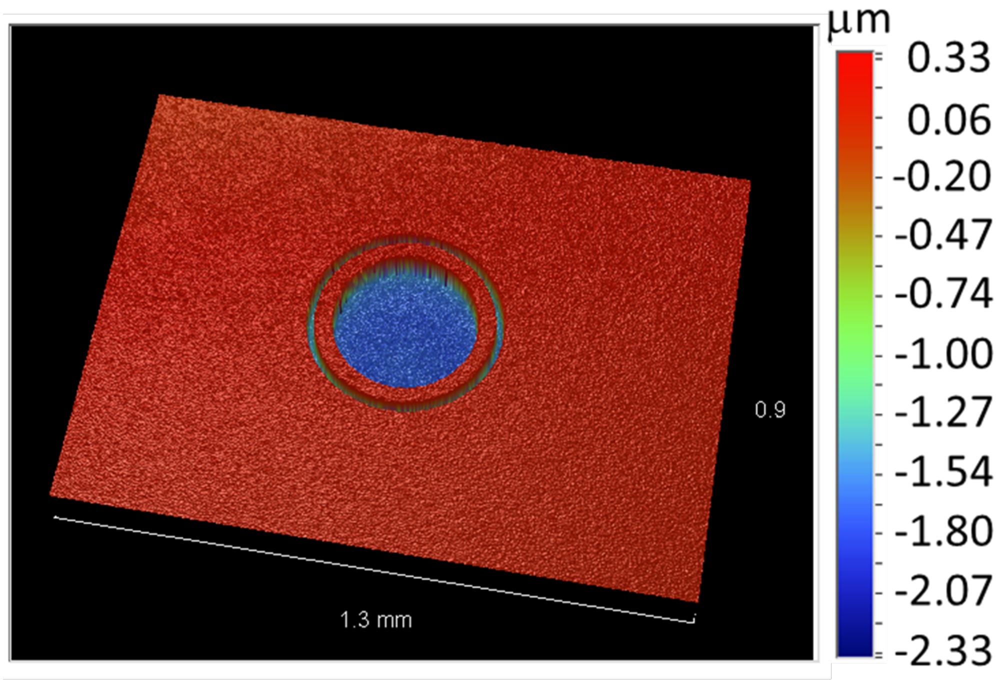

Figure S4 3D contour profiles of the inner region of the phase plate (notice that only the inner annular zones were measured due to the limited field of view of the instrument).
